# Unmasking the silent epidemic: a comprehensive systematic review and meta-analysis of undiagnosed diabetes in Ethiopian adults

**DOI:** 10.3389/fendo.2024.1372046

**Published:** 2024-07-17

**Authors:** Teshager Woldegiyorgis Abate, Ashenafi Genanew, Haileyesus Gedamu, Abebu Tegenaw, Emiru Ayalew, Alemeshet Yirga Berhie, Temesgen Ergetie, Belayneh Fentahun Shibesh

**Affiliations:** ^1^ Faculty of Nursing University of Alberta, Edmonton Clinic Health Academy, Edmonton, AB, Canada; ^2^ Department of Adult Health Nursing, Scholl of Health Sciences, College of Medicine and Health Sciences, Bahir Dar University, Bahir Dar, Ethiopia; ^3^ Department of Pharmacy, School of Health Sciences, College of Medicine and Health Sciences, Bahir Dar University, Bahir Dar, Ethiopia; ^4^ Department of Adult Health Nursing, School of Health Sciences, College of Medicine and Health Sciences, Bahir Dar University, Bahir Dar, Ethiopia; ^5^ Department of Psychiatry, School of Medicine, College of Medicine and Health Sciences, Bahir Dar University, Bahir Dar, Ethiopia; ^6^ Faculty of Medicine and Health Sciences, University of Oviedo, Oviedo, Spain; ^7^ Department of Public Health, Medical School of the University of Nicosia, Nicosia, Cyprus; ^8^ Nature, Climate and Health, United Nations University CRIS, Bruges, Belgium

**Keywords:** diabetes mellitus, undiagnosed diabetes, factors, meta-analysis, Ethiopia

## Abstract

**Background:**

Undiagnosed diabetes mellitus poses a significant global public health concern, exerting a substantial impact on the well-being of individuals, their families, and societies at large. Those individuals with undiagnosed diabetes miss opportunities to maintain quality of life and prevent diabetes-related complications. Even if there are ample primary studies on undiagnosed diabetes in Ethiopia, the results reveal conflicting results. Therefore, a comprehensive national picture of undiagnosed diabetes is essential for designing effective strategies at the national level.

**Methods:**

This study adhered to the Preferred Reporting Items for Systematic Reviews and Meta-Analyses guidelines for prevalence studies (PROSPERO ID: CRD42021266676). PubMed, Web of Science and the World Health Organization’s Hinari portal were searched using a strategy developed in collaboration with Liberians. The inclusion criteria comprised studies reporting undiagnosed diabetes in Ethiopia. Two independent reviewers conducted a quality assessment using a 10-item appraisal tool. Meta-analysis and meta-regression were performed using a random-effects model.

**Result:**

Twenty-five studies with 22,193 participants met the inclusion criteria. The pooled prevalence of undiagnosed diabetes among the Ethiopian adult population was 5.68% (95% CI: 4.53 - 6.83, I^2^ = 75.4). Factors significantly associated with undiagnosed diabetes include age, waist circumference, overweight, family history of diabetes, and a history of hypertension.

**Conclusion:**

Our systematic review found a noteworthy prevalence of undiagnosed diabetes in Ethiopia. The majority of factors linked with undiagnosed diabetes in this review were modifiable. This underscores the importance of targeted factors and public health interventions to improve early detection and reduce the burden of undiagnosed diabetes and its complications in Ethiopia.

**Systematic review registration:**

https://www.crd.york.ac.uk/prospero, identifier CRD42021266676.

## Background

Undiagnosed diabetes mellitus (UDM) occurs when an individual has diabetes, yet it remains unidentified by healthcare professionals, often due to a lack of medical testing or formal diagnosis (1). In the twenty-first century, nearly half of adults aged 20–79 with diabetes were unaware of their diabetic condition, comprising 44.7% (239.7 million individuals). Africa had the highest rates of UDM, with 53.6% of cases going unrecognized (2). Ethiopia, a country in the Sub-Sahara Africa, is experiencing a rapid epidemiological transition, marked by urbanization, lifestyle changes, and an increasing prevalence of non-communicable diseases parallel to communicable diseases (3, 4). Despite this, the determinants and prevalence of UDM in Sub-Saharan Africa, including Ethiopia, remain insufficiently understood, unlike communicable diseases (5, 6).

UDM is a major contributor to cardiovascular disease, kidney failure, neuropathy, nephropathy, vision impairment, and mental health issues, leading to various social and psychological challenges such as workplace difficulties, changes in family dynamics, an overall impact on quality of life, and death (7–13). Approximately 4.2 million deaths in the 20-79 age group are linked to diabetes, accounting for 11.3% of global deaths. Nearly half of these diabetes-related deaths (46.2%) occur in individuals under 60 years old. Notably, the Sub-Saharan African region bears the highest burden, with 73.1% of diabetes-related deaths occurring in individuals under the age of 60 years old in this region (14).

In resource-limited nations such as Ethiopia, the issue of UDM is alarming, with over half (54%) of affected individuals remaining undiagnosed (15). The epidemiological transition in these nations, characterized by rapid urbanization, urban poverty, and globalization, significantly contributes to the rising rates of UDM (16, 17). As populations shift from rural to urban settings, lifestyle changes and environmental factors play a role in the increasing prevalence of UDM (18).

UDM operates insidiously, acting as a silent threat due to the absence of noticeable symptoms in the early stages. This lack of early symptoms allows the disease to progress unnoticed, leading to severe complications that pose a significant threat to individual health. Moreover, these complications manifest as a socioeconomic burden, affecting both the afflicted individual and their family members (19, 20).

A critical barrier to early diagnosis is the limited diabetes knowledge (21) prevalent in resource-limited nations like Ethiopia (22). Insufficient awareness of symptoms, risk factors, and preventive measures hinders individuals from seeking timely medical attention (22). In addition to limited awareness, inadequate health facilities exacerbate the problem (20). The scarcity of well-equipped healthcare facilities (19, 23), coupled with a shortage of trained health workers, results in limited opportunities for individuals to undergo diabetes testing (22, 23). This scarcity not only contributes to delayed diagnoses but also perpetuates disparities in healthcare access, disproportionately affecting vulnerable populations (20, 24).

Despite a previous systematic review study conducted in Ethiopia (25), this study did not show the current status of UDM due to its small sample size and the omission of major primary studies conducted in Ethiopia. Within the domain of primary studies conducted in Ethiopia, a notable inconsistency and conflict of results emerge concerning UDM. This discrepancy underscores the imperative need for a comprehensive exploration and synthesis of existing research to reconcile and clarify the conflicting findings. This systematic review aims to bridge this knowledge gap by synthesizing existing literature and conducting a comprehensive meta-analysis. Through a rigorous examination of the available evidence, we seek to identify key factors associated with undiagnosed diabetes in the Ethiopian adult population, shedding light on areas that require targeted interventions and improved diagnostic strategies.

## Methods

### Protocol and registration

This study employs a systematic review and meta-analysis approach to estimate the prevalence of undiagnosed diabetes in Ethiopia. The reporting of the study adheres to the PRISMA (26) (Preferred Reporting Items for Systematic Reviews and Meta-Analyses) guidelines (27–29). To prevent duplication and uphold transparency, this review protocol was registered with the International Prospective Register of Systematic Reviews (PROSPERO) under the registration number CRD42021266676.

### Eligibility criteria

The inclusion criteria for this review were based on the Condition, Context, and Population (CoCoPo) framework, which considered studies focused on the prevalence of UDM and associated factors (conditions) in the Ethiopian (context) adult age group (population) (30). Both published and unpublished studies in the English language were considered. However, studies specifically addressing gestational diabetes, case reports, and qualitative designs were excluded from this review.

### Information source and search strategies

The search strategy was aligned with the systematic review question and inclusion criteria (CoCoPo). Two authors conducted a pretest of the PubMed database, and the actual electronic search was conducted between September 25 and October 5, 2023. Three reviewers systematically searched through PubMed, Web of Science, APA PsycInfo (EBSCO), CINAHL, MEDLINE, and Hinari, focusing on articles published in English between 2012 and 2023. Hinari, a World Health Organization (WHO) portal for low and middle-income countries, granted access to databases such as Web of Science, SCOPUS, AIM, CINAHL, IRIS, and African Journals Online. Although we did not find a study, grey literature was explored through institutional repositories, specifically Addis Ababa University and the WHO library. The search process was meticulously documented in a PRISMA flow chart, ensuring a systematic and thorough approach to literature identification (27).

For each key concept, relevant free-text words and MeSH terms were employed, and combinations were established using Boolean operators like “AND” and “OR.” This approach facilitated the retrieval of pertinent articles, accounting for different synonyms used in the literature. A search strategy was developed using fundamental concepts in the review questions: “prevalence,” “magnitude,” “burden,” “undiagnosed diabet*,” “diabet*,” “population,” “factor*,” “determinant*,” “influenc*,” “risk factor*,” “predictor*,” “Ethiopia” and English language (S1).

### Study selection

Following the search, all identified studies were imported into Endnote Desktop v.20 for efficient reference management and citation generation. Duplicate sources and publications that do not directly relate to the research question were removed. After removing duplication, the selection process followed the following process. Firstly, an initial pilot test of sample evidence sources was conducted to ensure clarity and consistency in applying inclusion and exclusion criteria. Following the pilot test, two independent reviewers (TWA and EA) screened the title and abstract against the inclusion criteria. Finally, the full text of the selected articles in the initial phase underwent a detailed assessment against the inclusion criteria by two additional independent reviewers (HG and AG). At any stage, disagreements between two reviewers were resolved through discussion and involving a third reviewer (BFS).

### Data collection process and quality assessment

The reviewer team employed a standardized data extraction form to extract the screening articles. The data extraction form was pilot-tested to assess the reliability and consistency of the extraction, as well as the appropriateness and usability of the form. Following pilot testing, two reviewers (TWA and EA) independently extracted data from papers included in the review using a finalized data extraction form (S2). The data extraction form included (but was not limited to) the first author, region, sample size, study period, study setting, study method and design, study purpose, outcome measurement tool, key findings as relevant to the review question (comorbid hypertension, overweight, smoking, family history of diabetes, sedentary behavior, and waist circumference), and participant demographic data (age). Disagreements in data abstraction between the first two reviewers were resolved by involving a third reviewer (BFS).

Given that the Newcastle–Ottawa Scale (NOS) (31) is a commonly used tool for appraising non-randomized studies (32), it was employed to assessed the quality of the included studies. The NOS comprises three categorical criteria (low, fair, and good) with a maximum score of ten points. This tool evaluates representatives of the sample, sample size, non-respondents, ascertainment of exposure, independent blind assessment, and statistical tests. Based on the NOS, we considered a study to have fair to good quality scores. Unfortunately, all primary studies were rated as fair to good quality (S3) (33–36).

### Outcome measurement

The primary outcome of this review was to determine the pooled prevalence of UDM among the adult population in Ethiopia. To analyze the second outcome (factors), relevant variables associated with UDM were extracted from each included article. In examining factors linked to the primary outcome, Adjusted Odd Ratios (AORs) from the primary studies were utilized to ascertain the association between independent variables and the presence of UDM.

### Risk assessment

The risk of bias in the included studies underwent assessment using a 10-item rating scale designed for prevalence studies (37). The assessment tool encompasses factors such as representative sample size, data collection method, reliability and validity of study tools, case definition, and prevalence periods of the studies. Each observational study article was classified based on a low risk of bias (affirmative responses to domain questions) or a high risk of bias (negative responses to domain questions). A score of 1 (Yes) or 0 (No) was assigned to each study for each domain, and these domain scores were aggregated to derive an overall study quality score. Scores falling within the range of 8–10 were deemed to indicate a “low risk of bias,” scores of 6–7 signified a “moderate risk,” and scores ranging from 0 to 5 were indicative of a “high risk.” In instances of disagreements between reviewers regarding the least risk of bias classification, resolution occurred through consensus.

### Data analysis

#### Testing for heterogeneity

Heterogeneity among the findings of the primary studies was assessed using Cochran’s Q test and was quantified through I-square statistics. A P-value of less than 0.1 suggested statistically significant heterogeneity. Heterogeneity was characterized by a small number of studies and their divergent designs (38). Classifications for heterogeneity were as follows: I-square values below 25% were considered lower, 25-75% were deemed moderate, and values above 75% were considered higher (39). Consequently, the random-effect model was applied to consolidate the prevalence of undiagnosed diabetes due to the identified heterogeneity in the studies (40). The random-effect model was utilized to investigate heterogeneity sources. Weighting in the meta-analysis accounted for residual between-study heterogeneity—heterogeneity not explained by the covariate in the regression (41). Publication bias was evaluated through visual inspection of funnel plots, subjectively assessing graph shape. A symmetrical graph suggested no publication bias, while asymmetry indicated its presence. Begg’s and Egger’s weighted regression were employed to assess publication bias objectively. P-values below 0.05 indicated significant publication bias (42, 43). Subgroup analyses by region and meta-regression using study year, sample size, and region were conducted due to significant heterogeneity among studies.

#### Statistical analysis

The data was analyzed in Stata Version 14, and presentation occurred in the evidence table, accompanied by summaries through descriptive statistics. The effect measure for outcome variables was computed utilizing the “Metaprop” command for meta-analysis of proportions in Stata. This review calculated the overall prevalence of UDM and its commonly associated factors alongside their respective 95% confidence intervals (CI). A forest plot was generated to depict the pooled prevalence of UDM and its common associated factors, including the author’s name, study year, and study weights, all at a 95% CI.

## Result

### Study selection process

**Figure 1:**
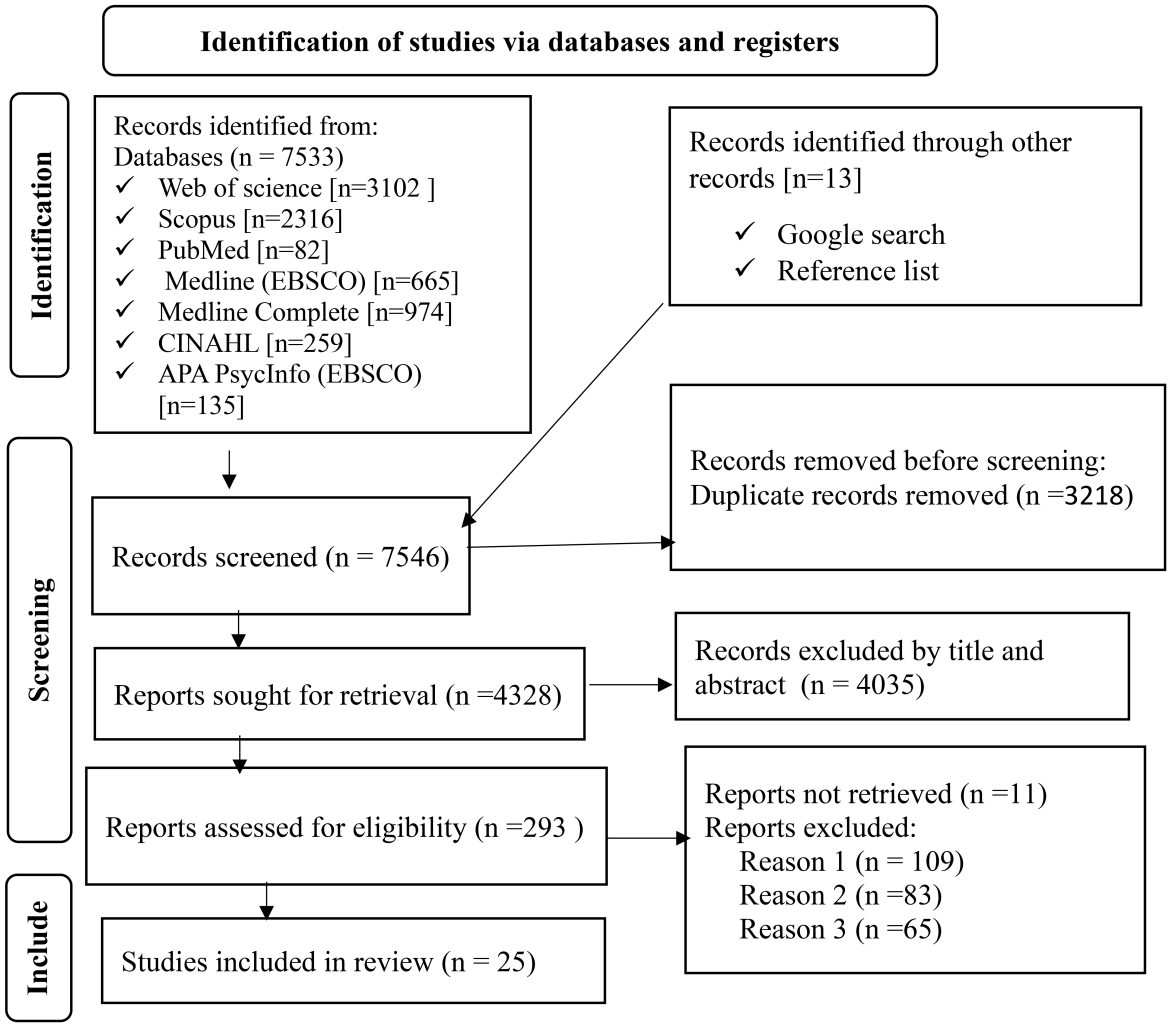
Flow chart to a selection of studies for a systematic review and meta-analysis of the prevalence of undiagnosed diabetes mellitus and associated factors among the Ethiopian adult population, Ethiopia, 2023.

After conducting a search in electronic databases and other records, a total of 7546 observational studies were identified, and 3218 were removed using Endnote V.20. Subsequently, out of the remaining 4328 studies, 4035 were excluded based on title and abstract screening. Out of the remaining 293 articles, 268 were excluded due to not meeting the inclusion criteria, such as differences in population, settings, and outcomes. Ultimately, 25 articles were included in the systematic review and meta-analysis (**Figure 1**).

### Study characteristics

In this systematic review and meta-analysis, we included 25 observational studies, encompassing 22,193 participants aged 15 years and older. The individual study cohorts ranged in size from 237 (44) to 4,371 (45) participants. The majority of the studies retrieved (n = 9) were conducted in the Amhara region (44, 46–53) followed by Southern Nations, Nationalities, and Peoples’ (SNNP) region of Ethiopia (n=7) (54–60), Oromia region (n=6) (45, 61–65), Addis Ababa (n=2) (66, 67), and Dire Dawa City Administration (n=1) (68).

All primary studies have utilized a lab-based cross-sectional design to estimate undiagnosed diabetes mellitus. Nearly all studies employed standardized blood glucose measurement tools and defined the outcome variable based on the diagnostic criteria established by the WHO, the American Diabetes Association (ADA), and the international diabetes federation (IDF). Six different measurement tools were used to assess blood glucose levels, with the glucose oxidase method being one of them. Additionally, five studies utilized the OneTouch Ultra Easy blood glucose meter (**Table 1**).

**Table 1:** Characteristics of studies included systematic review and meta-analysis, studies conducted from 2012–2023 among the Ethiopian adult population, Ethiopia, 2023.

Authors name	Study year	Source type	Region	Diagnostic criteria	Diagnostic method	SS	RR	PO	P	Quality score
Abebe SM. et al.	2012	J	Amhara	WHO and IDF	GO6-PD	1050	97	37	3.5	8
Animaw W. et al.	2015	J	Amhara	WHO	OTUEBGM	1405	95.5	35	2.5	6
Assefa A. et al.	2022	J	SNNP	IDF	OTUEBGM	415	98.3	61	14.7	8
Ayele BH. et al.	2017	J	DD	IDF	OTUEBGM	822	94	51	6.2	8
Aynalem SB. et al.	2016	J	SNNP	ADA	ACARD	402	97.1	23	5.75	8
Babtie GM. et al.	2018	J	Amhara	WHO	GO6-PD	607	100	62	10.2	8
Endris T. et al.	2019	J	Amhara	WHO	GO6-PD	587	98.2	29	4.9	8
Feyissa AA. et al.	2019	J	Oromia	WHO	H80CA	266	100	19	7.14	6
Damtie S. et al.	2021	J	Amhara	ADA	H80CA	407	100	18	4.5	8
Dereje N. et al.	2017	J	SNNP	WHO	ACARD	627	98.9	15	2.34	7
Jerene D. et al.	2021	J	AA	IDF	GO6-PD	2381	97	98	4	5
Hirigo AT. et al.	2019	J	SNNP	ADA	H80CA	237	96	15	6.3	7
Megerssa Y. et al.	2013	J	Oromia	WHO	GO6-PD	422	100	21	5	8
Seifu W. et al.	2009	J	Oromia	WHO	H80CA	4371	96.7	424	9.7	7
Teshome AA. et al.	2021	J	Amhara	ADA	H80CA	324	100	32	10	7
Wolde HF. et al.	2019	J	Amhara	WHO	GO6-PD	773	96	40	5.2	8
Woldesemayat B. et al.	2017	J	AA	WHO	OTUEBGM	392	92.9	10	2.6	7
Wondemagegn AT. et al.	2016	J	Amhara	WHO	GO6-PD	722	95.4	83	11.5	7
Wondemagegn AT. et al.	2016	J	Oromia	WHO	GO6-PD	530	98	46	8.67	8
Worede A. et al.	2015	J	Amhara	ADA	GO6-PD	392	100	9	2.3	7
Yohannes Seifu DT. et al.	2019	J	SNNP	WHO	OTUEBGM	516	100	48	9.3	7
Yunka TT. et al.	2019	J	Oromia	ADA	OTUEBGM	915	95.8	28	3.1	6
Zekewos A. et al.	2016	J	SNNP	ADA	GO6-PD	2670	98	55	2	7
Zenebe T. et al.	2017	J	Oromia	WHO	GO6-PD	264	98.1	15	5.7	5
Zenu S. et al.	2018	J	SNNP	WHO	i-QARE	696	97.2	86	12.3	8

PO, People with Outcome; J, Journal; GO6-PD, Glucose oxidase-6 phosphate dehydrogenase; WHO, World Health Organization; H80CA, Humastar 80 chemistry analyzer; OTUEBGM, ONETOUCH Ultra Easy Blood Glucose Meter; SS, Sample Size; NR, Not Reported; SNNP, Southern Nation Nationality People; AA, Addis Ababa; DD, Dire Dawa; IDF, International Diabetes Federation; ADA, American Diabetes Association.

### Quality appraisal

The quality score of the included study ranged from 5 to 8. Out of 25 studies, 17 (68%) received a low bias risk. Six studies (46, 47, 50, 55, 66, 68) had a high risk of case definition bias, and four studies (47, 51, 61, 66) had a high risk of representation bias (S4).

### Risk of bias

**Figure 2:**
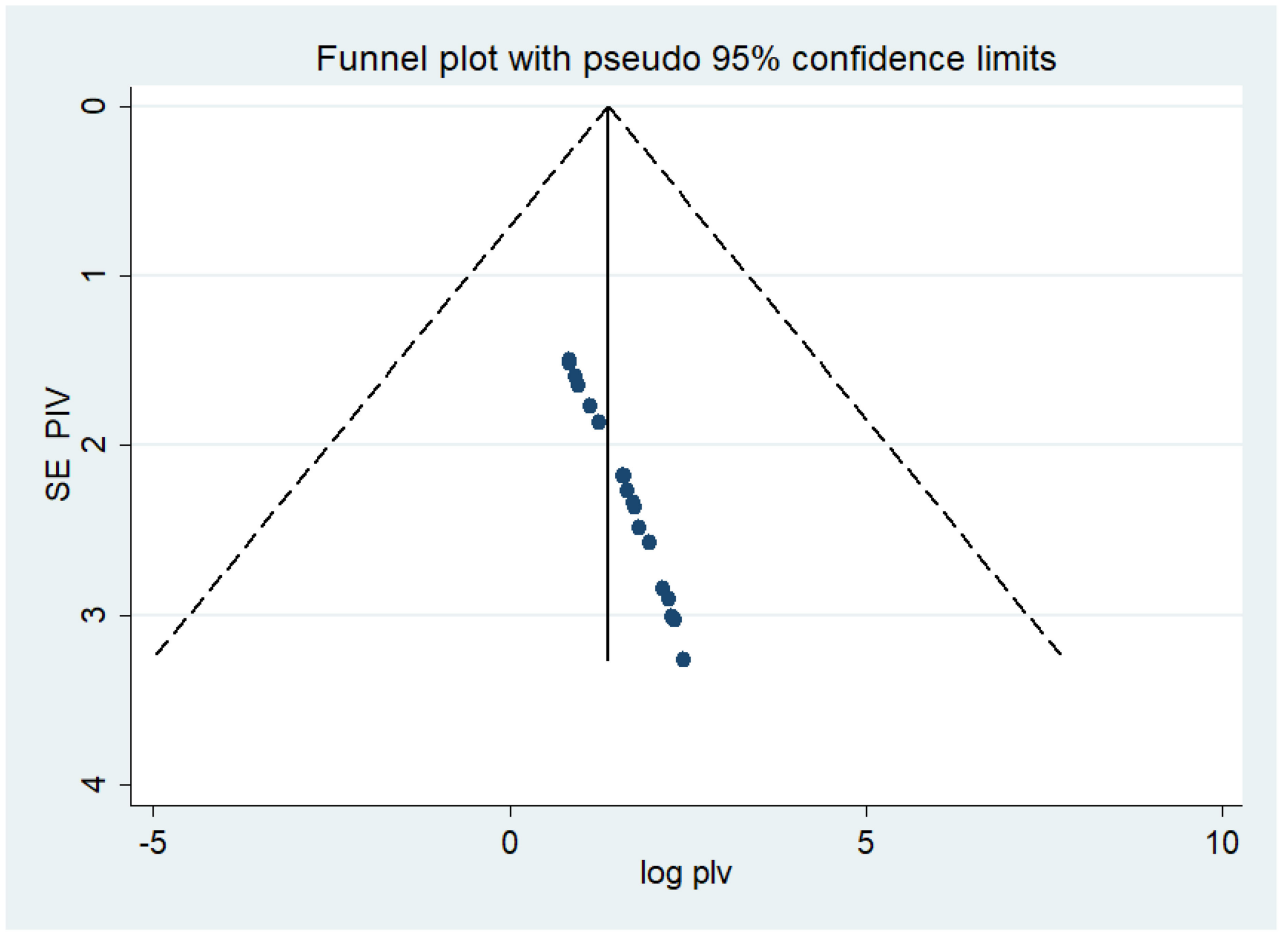
Meta funnel presentation of the prevalence of undiagnosed diabetes among the Ethiopian adult population, Ethiopia, 2023.

Both funnel plots of precision asymmetry and Egger’s intercept test revealed no publication bias in the primary studies. A visual examination of the funnel plot depicted asymmetry (**Figure 2**), and Egger’s test of the intercept (B0) yielded a value of 0.99 (95% CI: 0.94–1.04, p < 0.05). As judged by Egger’s test, no evidence of publication bias was presented at the 5% significance level.

### Meta-regression

#### Burden of undiagnosed diabetes mellitus in Ethiopian

**Figure 3:**
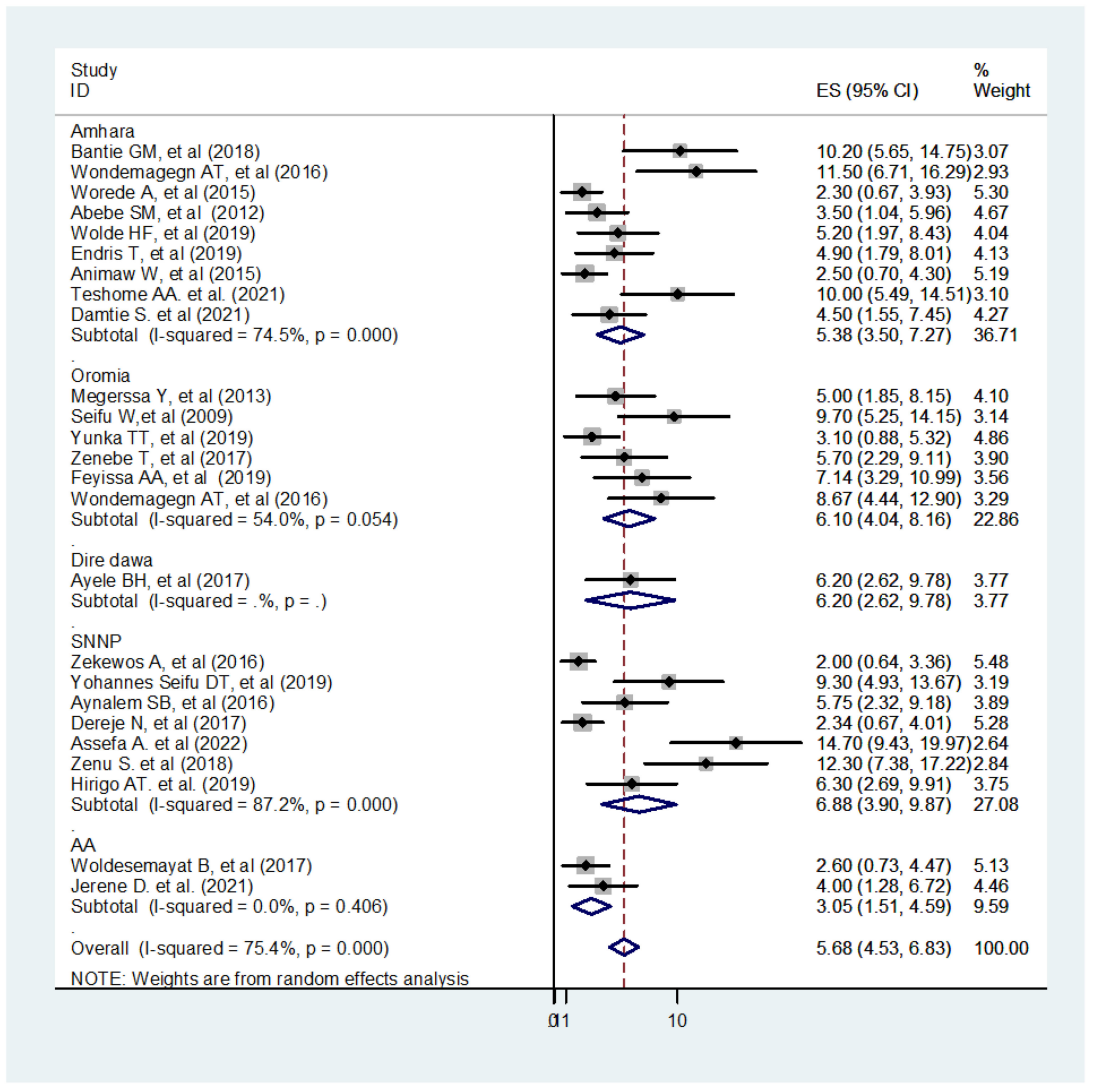
Forest plot of 25 studies assessing the prevalence of undiagnosed diabetes among the Ethiopian adult population, Ethiopia, 2023.

The analysis of 25 observational studies yielded rankings of low and moderate quality. In this study, the pooled prevalence of UDM in the Ethiopian adult population was 5.68% (95% CI: 4.53 - 6.83, I^2^ = 75.4%). Notably, there was borderline high heterogeneity among the studies included in the analysis, as indicated by the Q test (P<0.001) and I2 (I^2^ = 75.4%) (**Figure 3**). The I^2^ statistical test for heterogeneity revealed a significant variation between the studies (I^2^ = 75.4%, p < 0.05). Given the anticipated substantial differences in study settings and socio-economic contexts, a DerSimonian and Laird random-effects model was employed to estimate the pooled prevalence (69, 70). This model assigns weights to individual studies based on their reported effect size and sample size (38). To address the heterogeneity observed in the included studies, further subgroup analysis was conducted based on the regional location of undiagnosed diabetes. The highest and lowest pooled prevalence of undiagnosed diabetes were found in the SNNP regions and Addis Ababa city, with rates of 6.88% and 3.05%, respectively (**Figure 3**).

### Sub-group and meta-regression analysis

The sub-group analysis revealed a widespread heterogeneity across the studies (**Figure 3**). A meta-regression analysis were conducted to identify the source of this heterogeneity Subsequently, a meta-regression analysis was performed, incorporating the study year, sample size, and region as covariates. The results indicated that the listed covariates did not exhibit significance in the presence of heterogeneity across the studies (**Table 2**).

**Table 2:** Meta-regression output to explore the heterogeneity of the pooled prevalence of undiagnosed diabetes mellitus among the Ethiopian adult population, Ethiopia, 2023.

Variables	Coefficients	P-value
Study Year	-129.09	0.890
Sample size	3.18	0.073
Region		
Addis Ababa	1.63	0.756
Amhara	-1.47	0.672
Dire Dawa	Single study	Single study
Oromia	-0. 14	0.969
SNNPR	-1.26	0.734

### Determinants of undiagnosed diabetes mellitus in Ethiopia

Adjusted odds ratios obtained from primary studies were organized into three main categories: socio-demographic factors, anthropometric and clinical aspects, and behavioral perspectives. These categories were then integrated to identify the predominant risk factors associated with UDM within the adult population of Ethiopia. Commonly extracted adjusted odds ratios from the primary studies included variables such as age, waist circumference, overweight, family history of diabetes, history of hypertension, sedentary behaviors (physically inactive), and current smokers (**Table 3**).

**Table 3:** The relationship between undiagnosed diabetes mellitus and the pooled effect of factors eligible for meta-analysis among the Ethiopian adult population, Ethiopia, 2023.

Variables	Undiagnosed diabetes	
	POR (95% CI)	I^2^ (%)
Age more than 29 years old	2.07 (1.04, 3.10)	32.1
Wait circumference	6.32 (4.50, 8.15)	0.0
Overweight	2.05 (1.49, 2.61)	0.0
Family history of diabetes	2.98 (1.75, 4.22)	0.0
History of hypertension	4.80 (3.49, 6.11)	0.0
Sedentary behaviors (physically inactive)	2.36 (0.40, 4.32)	0.0*
Currently smoker	7.18 (-0.12, 14.48)	81.8*

*: Not statistically significant.

### Socio-demographic determinant of undiagnosed diabetes

The pooled effect reveals that individuals aged over 29 years are more than twice as likely to be exposed to UDM in the adult Ethiopian population (Pooled Odds Ratio (POR): 2.07; 95% CI: 1.04-3.10; I2 = 32.1%). This comprehensive review, encompassing eleven articles, consistently underscores the pivotal role of advancing age a significant determinant of UDM. The collective findings emphasize that as individuals age, they exhibit a heightened susceptibility to UDM, positioning age as a critical factor in understanding the prevalence and identification of this metabolic disorder.

In this review, the pooled effect of family history was pronounced threefold for UDM (POR: 2.98; 95% CI: 1.75-4.22; I^2^ = 0.0%). Family history emerges as a pivotal determinant of undiagnosed diabetes in Ethiopian adults, with 11 articles consistently highlighting its substantial predictive role. Demonstrating a hereditary component, individuals with a familial history of diabetes face elevated risks. The literature explores familial influence on disease dynamics, symptomatology, and healthcare-seeking behavior. These findings underscore the importance of integrating family history assessments into diabetes risk evaluations for tailored interventions in Ethiopia. Consistent evidence across multiple studies reinforces the necessity for healthcare practitioners to consider familial predisposition in enhancing preventive measures and early interventions for UDM (44, 46, 47, 49, 51, 53, 59, 63, 65–67).

### Anthropometric and clinical-related determinants of undiagnosed diabetes

The pooled odds ratio for individuals with a high waist circumference showed a more than sixfold increase in the vulnerability to UDM when compared to those with a lower waist circumference (Pooled Odds Ratio (POR): 6.32; 95% Confidence Interval (CI): 4.50-8.15; I2 = 0.0%). This comprehensive review, encompassing seven studies, consistently highlighted high waist circumference as a significant determinant of undiagnosed UDM (53–57, 61, 62, 66).

In this comprehensive review, overweight emerged as a significant determinant of UDM. The analysis revealed that individuals classified as overweight were twice as likely to develop UDM compared to those with a healthy body weight (POR: 2.05; 95%CI: 1.49-2.61; I2 = 0.0%). Eleven primary studies consistently highlighted overweight as a significant risk factor for UDM. These findings underscore the crucial role of excess weight as a substantial risk factor in UDM development, emphasizing the importance of weight management strategies in diabetes prevention efforts (44, 46, 48, 51, 52, 55–57, 63, 64, 68).

The pooled effect of fifteen studies revealed that individuals with a history of hypertension face a more than fourfold increased risk of UDM compared to those without a history of hypertension (POR: 4.80; 95%: 3.49-6.11; I^2^ = 0.0%). This significant association was consistently observed across fifteen primary studies, collectively indicating that a history of hypertension stands out as the predominant determinant in the development of UDM. The robust findings from these studies emphasize the critical role of hypertension history as a key risk factor for UDM, recommending the necessity for targeted interventions and vigilant monitoring in individuals with a hypertensive background to prevent and manage the onset of UDM (44, 47, 48, 51–59, 61, 64, 66).

### Behavioral determinates of undiagnosed diabetes

Physically inactive and smoking habits were identified as significant determinants of UDM in the primary studies. According to seven primary studies, individuals with physically inactive behaviors were more likely at risk for UDM compared to those adopting physically active behaviors. The consistent findings across these studies emphasize a significant association between sedentary habits and an increased likelihood of UDM. This underscores the importance of promoting physical activity and discouraging prolonged periods of inactivity to mitigate the risk of Undiagnosed Diabetes Mellitus among the studied population (45, 47, 57–60, 65).

Three primary studies conducted a thorough investigation into the complex relationship between smoking habits and the onset of UDM. These studies not only confirmed a correlation between smoking and UDM but also emphasized the need for a detailed exploration to uncover the specific impact of smoking on the likelihood of UDM occurrence. This nuanced approach sought to achieve a comprehensive understanding of how smoking, as a distinct lifestyle factor, contributes to the intricate landscape of diabetes (45, 51, 55).

However, the pooled effect did not indicate a significant association between sedentary behaviors (POR: 2.36; 95% CI: 0.40 to 4.32, I^2^ = 0.0%) and smoking habits (POR: 7.18; 95% CI: -0.12 to 14.48, I^2^ = 81.8%). Notably, the absence of a significant association in the pooled effect does not necessarily imply that smoking and sedentary behavior do not pose a risk for UDM.

## Discussion

This systematic review and meta-analysis aimed to determine the pooled prevalence of UND and identify common determinants among the adult population in Ethiopia. The study revealed a high prevalence of UDM (5.68%) among the Ethiopian adult population compared to a systematic review done in the African region (71). Additionally, regional variations in UDM were observed in this systematic review, with the highest rates reported in SNNP (6.88%), followed by Oromia (6.10%) and Amhara (5.38%), while the lowest prevalence was recorded in Addis Ababa (3.05%). The identified prevalence in this study exceeds that reported in a systematic meta-analysis conducted in Africa (3.85%) (71). This higher figure may suggest a lack of awareness regarding diabetes symptoms and a limited understanding, attitude, and adherence to diabetes screening practices (72–74). Contributing factors could include limited healthcare access, insufficient nationwide screening programs (19, 75, 76), and rapid epidemiological changes. These changes, characterized by unhealthy lifestyles marked by poor dietary habits, insufficient physical activity, and rising obesity rates, may collectively contribute to the increased prevalence of UDM (77, 78).

The prevalence of UDM in our study aligns closely with rates reported in a study conducted in Canada (79), and another in Qatar (80). This concordance may be attributable to the fact that diabetes detection remains inadequate globally (81), and asymptomatic nature, where individuals may not exhibit noticeable symptoms, leading to underdiagnosis across different geographical regions (82). The similarity in prevalence rates across diverse populations underscores the need for heightened awareness and improved screening strategies to identify cases of UDM that might otherwise go unnoticed due to the absence of overt symptoms (82, 83).

This finding surpasses the prevalence reported in a study conducted in Sudan (84) but is lower than those observed in studies conducted in Kuwait (85), Hong Kong (86), India (87), Iraq (88), Australia (89), and Texas (90). The observed discrepancy may stem from both systemic and personal factors. System challenges such as poor access to screening and basic diagnostic tools in primary healthcare settings, population changes, western cultural influences, and low-quality healthcare could contribute (17). On a personal level, factors like poverty and cost, educational status, and perceptions about the disease may also play a role (91).

The higher prevalence of UDM observed in Sub-Saharan Africa, including Ethiopia, underscores the inadequacy of guidelines for the screening and diagnosis of diabetes, as well as the insufficiency of trained staff in the primary health service setting (92). This suggests that there is a notable gap in the resources and infrastructure necessary for effectively addressing diabetes within the primary healthcare system (19, 22, 23).

The lower prevalence of UDM in Sudan can be attributed to various interconnected factors. Diabetes receives low priority in the healthcare system due to a lack of dedicated diabetes care centers and insufficiently trained personnel (93). There is also a lack of educational initiatives for patients and limited public awareness regarding diabetes in Sudan (94).

The high UDM in the Australian experience has led the national authorities to endorse a national evidence-based guideline for the case detection and diagnosis of diabetes. This guideline recommends a stepped approach to diagnosing individuals with previous UDM based on an individual’s risk status assessment. It involves measuring fasting plasma glucose in individuals at risk, and further testing is required according to the fasting plasma glucose result (95).

One potential reason for the elevated prevalence of UDM in Texas may be attributed to the aggressive detection of UDM. For example, Texas state agencies actively participate in numerous programs dedicated to preventing, screening, and treating diabetes (96).

Therefore, halting the rise in diabetes is achieved through the availability of diagnosed guidelines, training for healthcare staff, allocation of budgetary resources, and prioritizing diabetes within primary healthcare. Reducing the burden of diabetes involves strengthening preventive strategies that align with the acceptable local socio-cultural context, representing a cost-effective approach (97).

In this review, factors such as age, waist circumference, overweight, family history of diabetes, and a history of hypertension were significantly associated with UDM in the Ethiopian adult population. Age emerges as a potential factor influencing UDM. The recurrent report of age in the primary literature underscores its complex link to a higher likelihood of UDM. According to the evidence presented, advanced group individuals face a distinct risk profile due to age-related physiological changes and potential physical barriers to healthcare access. It underscores the need for healthcare professionals to consider age as a key factor in designing interventions, screening programs, and healthcare policies related to diabetes. Furthermore, gaining insights into how age influences the dynamics of undiagnosed diabetes can contribute to developing more targeted and effective public health strategies tailored to the unique challenges older individuals face in the context of diabetes detection and management. Understanding the socio-demographic determinants of undiagnosed diabetes is crucial for designing targeted interventions and public health strategies aimed at improving early detection and management. Tailoring healthcare initiatives to address these determinants can contribute to reducing the burden of undiagnosed diabetes within specific population groups (45, 47, 49, 50, 52, 54, 59, 60, 62, 65, 66, 68).

Being age advanced increased susceptibility with advancing age can be attributed to a decline in insulin sensitivity and alterations or insufficient compensation of beta-cell function in response to escalating insulin resistance, ultimately leading to diabetes (98). The deterioration in glucose tolerance from young (17–39 years) to middle age (40–59 years) is elucidated by the secondary influences of body fat and physical fitness, encompassing factors such as obesity, central and upper-body fat deposition, and physical inactivity (99).

The evidence showed that age directly affects β-cell proliferation and function, while also indirectly contributing to impaired insulin sensitivity through lifestyle-related and comorbidity-related risk factors. Insulin resistance, in turn, may exacerbate β-cell dysfunction (100). Lifestyle modifications, including adopting healthy dietary habits and engaging in physical activity to reduce obesity, play a crucial role in preventing the early onset of diabetes by decreasing insulin resistance and increasing beta-cell volume (101). Preventing new-onset diabetes not only reduces the risk of microvascular diseases but also lowers healthcare costs and enhances health-related quality of life (102).

Given that age is a significant diabetes risk factor, screening is recommended to commence no later than age 45 for all individuals. Moreover, it should be considered in adults of any age with overweight or obese and one or more risk factors for diabetes (103). Addressing age-related factors, adopting a healthy lifestyle, and timely screening are essential strategies for preventing diabetes, reducing associated complications, and improving overall health outcomes (104).

The result of this review shown that being overweight as a determinant of UDM, aligning with studies in India (105), Kuwaiti (85), and Qatar (80) linking increased body mass index (BMI) to higher UDM prevalence. Being overweight disrupts cellular mechanisms, leading to insulin resistance, characterized by reduced liver, muscle, and adipose tissue responsiveness in type 2 diabetes (106). This disruption is explained by the essential role of stored fat in survival during nutritionally deprived states. Excessive fat storage, leading to obesity, triggers elevated fatty acids (FFAs) release through enhanced lipolysis, contributing to insulin resistance (107). This fatty acid did not only reduce the utilization of insulin-stimulated muscle glucose, exacerbating hyperglycemia, but also lead to lipotoxicity. This lipotoxicity hampers the secretion of insulin by pancreatic β-cells, eventually causing β-cell exhaustion and the development of diabetes (106, 108–111). Consequently, incorporating risk-based screening into health services, particularly in developing countries, is crucial (103). Testing is recommended for adults with overweight or obesity (BMI >25 kg/m2) as they are particularly at risk (112).

In this review, the family history of diabetes emerged as another determinant of UDM in the Ethiopian adult population. Existing research indicates that the risk of diabetes diagnosis increases significantly, ranging from two- to fourfold when one or both parents are lived with diabetes (113–115). Because of this, the American Diabetes Association standard of care recommended that testing should be considered after the onset of puberty or after 10 years of age in first-degree relative with diabetes (112). Moreover, individuals with co-morbid hypertension in Ethiopia exhibit an increased susceptibility to developing UDM. Diabetes mellitus and hypertension manifest as prevalent diseases, coexisting at a frequency higher than mere chance would suggest (116). This observation aligns with previous research that identifies hypertension as a common risk factor for diabetes (117, 118).

The progression of hypertension and diabetes mellitus demonstrates a parallel trajectory over time, with insulin resistance serving as a shared feature in both pre-diabetes and prehypertension, acting as a precursor to the eventual manifestation of the two respective diseases (119). In a broader context, the foundation for preventing and managing diabetes lies in lifestyle behavior modification. This entails incorporating regular physical exercise, effective weight management, and adherence to healthcare professionals’ recommendations regarding a healthy diet (91, 120).

### Strengths and limitation

This systematic review and meta-analysis possess notable strengths. It is comprehensive, pooling the results of various studies in the country to offer stronger evidence regarding different factors associated with UDM among the adult population in Ethiopia. The review incorporated a relatively large number of individuals with diabetes (N = 22,193), surpassing the sample sizes of individual studies. Moreover, it systematically investigated numerous factors associated with UDM and presented insightful suggestions. Despite these strengths, the study is not without limitations. While most of the included studies demonstrated good quality, it is crucial to note that all primary studies were cross-sectional, limiting the depth of this analysis. Additionally, despite employing extensive and diverse search strategies to uncover all possible relevant literature, some grey literature, such as conference proceedings, proved challenging to locate.

### Implication

This review on UDM in Ethiopian adults has far-reaching implications for public health and healthcare strategies. The findings illuminate the prevalence and determinants of UDM, prompting crucial considerations for various facets of intervention. Firstly, heightened public health awareness campaigns are urgently needed to address undiagnosed diabetes. Educational programs are essential to inform the Ethiopian population about risks, symptoms, and consequences, fostering early detection and management. Additionally, the study underscores the necessity to fortify healthcare infrastructure, specifically in terms of diabetes screening and diagnostic facilities. Investments in accessible tools and healthcare professional training are imperative for effective undiagnosed diabetes management.

Policymakers in Ethiopia are urged to prioritize early diabetes screening, suggesting the integration of routine screenings into primary healthcare services as a key strategy for prompt identification. The comprehensive nature of the review emphasizes the importance of continued research efforts, advocating for collaboration between researchers, healthcare institutions, and governmental bodies. Lastly, evidence-based public health interventions tailored to the Ethiopian context, such as community-based screenings and lifestyle modification programs, are deemed necessary to prevent and manage diabetes effectively. This holistic approach underscores the multidimensional response required to address the challenges posed by undiagnosed diabetes in Ethiopian adults.

## Conclusion

Our systematic review and meta-analysis revealed a noteworthy prevalence of UDM among the adult population in Ethiopia, with an average pooled prevalence of 5.68%. The identified factors associated with UDM, including age, increased waist circumference, overweight, family history of diabetes, and a history of hypertension, highlight the complex interplay of various determinants contributing to the underdiagnoses of diabetes in this population.

## Data availability statement

The original contributions presented in the study are included in the article/**Supplementary Material**. Further inquiries can be directed to the corresponding author.

## Author contributions

TA: Conceptualization, Data curation, Formal analysis, Methodology, Validation, Writing – original draft, Writing – review & editing. AG: Methodology, Visualization, Writing – review & editing. HG: Methodology, Validation, Visualization, Writing – review & editing. AT: Methodology, Validation, Visualization, Writing – review & editing. EA: Methodology, Validation, Visualization, Writing – review & editing. AB: Methodology, Validation, Visualization, Writing – review & editing. TE: Methodology, Validation, Visualization, Writing – review & editing. BS: Methodology, Visualization, Writing – review & editing.
