# Supplementary material for: Unmasking the silent epidemic: a comprehensive systematic review and meta-analysis of undiagnosed diabetes in Ethiopian adults
Source: Front Endocrinol (Lausanne). 2024 Jul 17;15:1372046. doi: 10.3389/fendo.2024.1372046 (PMC11288971; doi:10.3389/fendo.2024.1372046)
Supplement: Supplementary file 1 [file DataSheet_1.zip › S1.pdf]

S1. Search string with the output

Database	Search string	No. of article
PubMed	(("epidemiology"[MeSH Subheading] OR "epidemiology"[All Fields] OR "prevalence"[All Fields] OR "prevalence"[MeSH Terms] OR "prevalance"[All Fields] OR "prevalences"[All Fields] OR "prevalence s"[All Fields] OR "prevalent"[All Fields] OR "prevalently"[All Fields] OR "prevalents"[All Fields] OR ("magnitude"[All Fields] OR "magnitudes"[All Fields]) OR ("burden"[All Fields] OR "burdened"[All Fields] OR "burdening"[All Fields] OR "burdens"[All Fields])) AND ("systematic review"[Filter] AND "english"[Language]) AND (("undiagnosed diabet*"[All Fields] OR "diabet*"[All Fields]) AND ("systematic review"[Filter] AND "english"[Language])) AND (("factor*"[All Fields] OR "determinant*"[All Fields] OR "influenc*"[All Fields] OR "predictor*"[All Fields]) AND ("systematic review"[Filter] AND "english"[Language])) AND (("ethiopia"[MeSH Terms] OR "ethiopia"[All Fields] OR "ethiopia s"[All Fields]) AND ("systematic review"[Filter] AND "english"[Language]))) AND ((2012:2023[pdat]) AND (english[Filter]))	82
Medline (EBSCO)	(prevalence OR magnitude OR burden) AND ("undiagnosed diabet*" OR diabet* OR population) AND (factor* OR determinant* OR influenc* OR predictor*) AND Ethiopia	665
Medline Complete (EBSCO)	(prevalence OR magnitude OR burden) AND ("undiagnosed diabet*" OR diabet* OR population) AND (factor* OR determinant* OR influenc* OR predictor*) AND Ethiopia	974
CINAHL	(prevalence OR magnitude OR burden) AND ("undiagnosed diabet*" OR diabet* OR population) AND (factor* OR determinant* OR influenc* OR predictor*) AND Ethiopia	259
APA PsycInfo (EBSCO)	(prevalence OR magnitude OR burden) AND ("undiagnosed diabet*" OR diabet* OR population) AND (factor* OR determinant* OR influenc* OR predictor*) AND Ethiopia	135
SCOPUS	(TITLE-ABS-KEY (prevalence OR magnitude OR burden) AND TITLE-ABS-KEY ({undiagnosed diabet} OR diabet* OR population) AND TITLE-ABS-KEY (factor* OR determinant* OR influenc* OR predictor*) AND TITLE-ABS-KEY (ethiopia)) AND PUBYEAR > 2008 AND PUBYEAR < 2024 AND (LIMIT-TO (LANGUAGE , "English")) AND (LIMIT-TO (DOCTYPE , "ar") OR LIMIT-TO (DOCTYPE , "re"))	2,316
Web of Science	https://www.webofscience.com/wos/alldb/summary/6b50cc9f-6c76-4688-a73f-528ddc87f81d-e973c7ff/relevance/1	3102
