# Supplementary material for: Unmasking the silent epidemic: a comprehensive systematic review and meta-analysis of undiagnosed diabetes in Ethiopian adults
Source: Front Endocrinol (Lausanne). 2024 Jul 17;15:1372046. doi: 10.3389/fendo.2024.1372046 (PMC11288971; doi:10.3389/fendo.2024.1372046)
Supplement: Supplementary file 1 [file DataSheet_1.zip › S3.docx]

**S3. Scoring of the Quality of Articles by Authors using the Newcastle-Ottawa Quality Assessment Tool.**

| Studies by authors name | Quality assessors | Selection | | | | Comparability | | Outcome | | Total score |
| --- | --- | --- | --- | --- | --- | --- | --- | --- | --- | --- |
|  |  | Representativeness of sample (*, *) | Sample size (*) | Non-respondents (*) | Ascertainments (**) | Study control for most important factors (*) | The study controls any additional factors (*) | Assessment of the outcome (*) | Statistical test (*) |  |
| Abebe SM. et al. | TE | 1 | 1 | 1 | 1 | 0 | 1 | 1 | 1 | 6 |
|  | TWA | 1 | 0 | 1 | 1 | 1 | 0 | 1 | 1 |  |
|  | AG | 2 | 1 | 0 | 1 | 1 | 1 | 0 | 1 |  |
| Animaw W. et al. | HG | 1 | 1 | 1 | 1 | 0 | 1 | 1 | 1 | 7 |
|  | TE | 1 | 0 | 0 | 2 | 1 | 1 | 0 | 1 |  |
|  | TWA | 1 | 1 | 1 | 0 | 0 | 1 | 1 | 1 |  |
| Ayele BH. et al. | AG | 1 | 1 | 1 | 1 | 1 | 1 | 0 | 1 | 8 |
|  | HG | 1 | 0 | 1 | 1 | 0 | 1 | 1 | 0 |  |
|  | TE | 1 | 1 | 1 | 0 | 1 | 0 | 1 | 1 |  |
| Aynalem SB. et al. | TWA | 1 | 0 | 1 | 1 | 0 | 1 | 1 | 0 | 8 |
|  | AG | 1 | 1 | 1 | 1 | 1 | 1 | 1 | 1 |  |
|  | HG | 0 | 0 | 1 | 1 | 0 | 1 | 1 | 1 |  |
| Babtie GM. et al. | TE | 2 | 1 | 0 | 1 | 1 | 1 | 1 | 1 | 6 |
|  | TWA | 2 | 1 | 1 | 1 | 1 | 0 | 1 | 1 |  |
|  | AG | 1 | 1 | 0 | 1 | 1 | 1 | 1 | 1 |  |
| Endris T. et al. | AG | 1 | 1 | 1 | 1 | 1 | 1 | 1 | 1 | 6 |
|  | HG | 1 | 1 | 0 | 1 | 1 | 1 | 1 | 1 |  |
|  | TE | 1 | 0 | 0 | 1 | 1 | 0 | 1 | 1 |  |
| Feyissa AA. et al. | HG | 2 | 1 | 1 | 1 | 1 | 1 | 1 | 1 | 8 |
|  | TE | 1 | 0 | 1 | 0 | 1 | 1 | 0 | 1 |  |
| Endris T. et al. | TWA | 1 | 1 | 1 | 1 | 1 | 1 | 1 | 1 |  |
|  | AG | 1 | 1 | 1 | 1 | 1 | 1 | 1 | 1 | 6 |
|  | HG | 1 | 1 | 0 | 1 | 1 | 1 | 1 | 1 |  |
|  | TE | 1 | 0 | 0 | 1 | 1 | 0 | 1 | 1 |  |
| Damtie S.et al. | TWA | 1 | 1 | 1 | 1 | 0 | 1 | 0 | 1 | 8 |
|  | AG | 0 | 1 | 1 | 1 | 1 | 1 | 1 | 1 |  |
|  | HG | 1 | 0 | 1 | 1 | 0 | 1 | 1 | 1 |  |
| Dereje N. et al. | TWA | 1 | 1 | 1 | 1 | 0 | 1 | 0 | 1 | 8 |
|  | AG | 0 | 1 | 1 | 1 | 1 | 1 | 1 | 1 |  |
|  | HG | 1 | 0 | 1 | 1 | 0 | 1 | 1 | 1 |  |
| Jerene D.et al. | AG | 1 | 1 | 1 | 1 | 1 | 1 | 1 | 1 | 6 |
|  | HG | 1 | 1 | 0 | 1 | 1 | 1 | 1 | 1 |  |
|  | TE | 1 | 0 | 0 | 1 | 1 | 0 | 1 | 1 |  |
| Hirigo At. et. al | TWA | 1 | 1 | 1 | 1 | 0 | 1 | 0 | 1 | 8 |
|  | AG | 0 | 1 | 1 | 1 | 1 | 1 | 1 | 1 |  |
|  | HG | 1 | 0 | 1 | 1 | 0 | 1 | 1 | 1 |  |
| Megerssa Y. et al. | TE | 1 | 1 | 0 | 1 | 1 | 1 | 1 | 1 | 7 |
|  | TWA | 1 | 0 | 1 | 1 | 0 | 0 | 1 | 1 |  |
|  | AG | 1 | 1 | 0 | 1 | 1 | 1 | 1 | 1 |  |
| Seifu W.et al. | HG | 2 | 1 | 1 | 1 | 1 | 1 | 1 | 1 | 8 |
|  | TE | 1 | 1 | 1 | 1 | 1 | 1 | 1 | 1 |  |
|  | TWA | 2 | 1 | 1 | 1 | 0 | 1 | 1 | 1 |  |
| Wolde HF. et al. | AG | 1 | 1 | 0 | 1 | 1 | 1 | 1 | 1 | 7 |
|  | HG | 2 | 1 | 1 | 1 | 1 | 1 | 1 | 1 |  |
|  | TE | 1 | 1 | 1 | 1 | 1 | 1 | 1 | 1 |  |
| Woldesemayat B. et al. | TWA | 0 | 1 | 1 | 1 | 0 | 0 | 0 | 1 | 8 |
|  | AG | 1 | 0 | 0 | 0 | 1 | 1 | 1 | 0 |  |
|  | HG | 1 | 1 | 1 | 1 | 1 | 1 | 1 | 1 |  |
| Wondemagegn AT. et al. | TE | 2 | 1 | 0 | 1 | 1 | 1 | 1 | 1 | 7 |
|  | TWA | 2 | 1 | 1 | 1 | 1 | 1 | 1 | 1 |  |
|  | AG | 1 | 1 | 0 | 0 | 1 | 1 | 1 | 1 |  |
| Wondemagegn AT. et al. | HG | 2 | 1 | 1 | 1 | 1 | 1 | 1 | 1 | 7 |
|  | TE | 2 | 1 | 1 | 1 | 1 | 1 | 1 | 1 |  |
|  | TWA | 1 | 0 | 1 | 1 | 1 | 1 | 1 | 1 |  |
| Worede A. et al. | AG | 2 | 1 | 1 | 1 | 1 | 1 | 1 | 1 | 7 |
|  | HG | 1 | 1 | 1 | 1 | 1 | 1 | 1 | 1 |  |
|  | TE | 2 | 1 | 1 | 1 | 1 | 1 | 1 | 1 |  |
| Yohannes Seifu DT. et al. | TWA | 2 | 1 | 1 | 1 | 0 | 1 | 1 | 1 | 7 |
|  | AG | 1 | 1 | 0 | 1 | 1 | 1 | 1 | 1 |  |
|  | HG | 1 | 1 | 1 | 1 | 1 | 1 | 1 | 1 |  |
| Yunka TT. et al. | TE | 1 | 1 | 1 | 1 | 1 | 1 | 1 | 1 | 8 |
|  | TWA | 1 | 0 | 1 | 1 | 0 | 0 | 0 | 1 |  |
|  | AG | 1 | 1 | 0 | 0 | 1 | 0 | 1 | 0 |  |
| Yunka TT. et al. | TE | 1 | 1 | 1 | 1 | 1 | 1 | 1 | 1 | 8 |
|  | TWA | 1 | 0 | 1 | 1 | 0 | 0 | 0 | 1 |  |
|  | AG | 1 | 1 | 0 | 0 | 1 | 0 | 1 | 0 |  |
| Zekewos A. et al. | HG | 2 | 1 | 1 | 1 | 0 | 1 | 1 | 1 | 7 |
|  | TE | 1 | 1 | 0 | 1 | 1 | 1 | 1 | 1 |  |
|  | TWA | 1 | 1 | 1 | 1 | 1 | 1 | 1 | 1 |  |
| Zenebe T. et al. | HG | 1 | 1 | 1 | 1 | 1 | 1 | 1 | 1 | 7 |
|  | TE | 1 | 1 | 1 | 1 | 1 | 1 | 1 | 1 |  |
|  | TWA | 1 | 0 | 1 | 0 | 0 | 1 | 0 | 1 |  |
| Zenu S. et al. | HG | 1 | 1 | 1 | 1 | 1 | 1 | 1 | 1 | 8 |
|  | TE | 1 | 1 | 1 | 1 | 1 | 1 | 1 | 1 |  |
|  | TWA | 1 | 0 | 1 | 0 | 0 | 1 | 0 | 1 |  |
